# Female sexual dysfunction: prevalence and risk factors in a cohort of women living with HIV in Italy

**DOI:** 10.1093/sexmed/qfaf038

**Published:** 2025-05-19

**Authors:** Martina Salvi, Giorgio Tiecco, Maria Alberti, Francesco Castelli, Emanuele Focà, Eugenia Quiros-Roldan

**Affiliations:** Department of Clinical and Experimental Sciences, Unit of Infectious and Tropical Diseases, University of Brescia and ASST Spedali Civili di Brescia, 25123 Brescia, Italy; Department of Clinical and Experimental Sciences, Unit of Infectious and Tropical Diseases, University of Brescia and ASST Spedali Civili di Brescia, 25123 Brescia, Italy; Department of Clinical and Experimental Sciences, Unit of Infectious and Tropical Diseases, University of Brescia and ASST Spedali Civili di Brescia, 25123 Brescia, Italy; Department of Clinical and Experimental Sciences, Unit of Infectious and Tropical Diseases, University of Brescia and ASST Spedali Civili di Brescia, 25123 Brescia, Italy; Department of Clinical and Experimental Sciences, Unit of Infectious and Tropical Diseases, University of Brescia and ASST Spedali Civili di Brescia, 25123 Brescia, Italy; Department of Clinical and Experimental Sciences, Unit of Infectious and Tropical Diseases, University of Brescia and ASST Spedali Civili di Brescia, 25123 Brescia, Italy

**Keywords:** HIV, female sexual dysfunction, women, FSFI

## Abstract

**Background:**

Female sexual dysfunction (FSD) has an impact on the lives of many women, and it is inadequately investigated by medical professionals in women living with HIV (WLWH).

**Aim:**

In the present study, the aim was to investigate the prevalence and risk factors of sexual dysfunction (SD) in a cohort of WLWH using the Female Sexual Function Index (FSFI) questionnaire.

**Methods:**

This monocentric cross-sectional study was conducted at the ASST Spedali Civili of Brescia, Italy, between April 2023 and August 2023. To assess SD, the FSFI questionnaire was administered in accordance with current European AIDS Clinical Society guidelines to all consecutive cisgender adult WLWH who presented to our unit of Infectious Diseases. We used FSFI < 26.55 to identify participants at risk of SD. Participants were divided into two groups based on the pathological cut-off of FSFI score.

**Outcome:**

Comparison of demographic characteristics, menopausal status, and comorbidities among the two different groups of WLWH.

**Results:**

The questionnaire was offered to 371 women and 179 (48.2%) completed it. Of the 192 (51,8%) excluded, there were 129 women who declined to participate and 63 who were unable to do so due to a language barrier. Two-thirds (117/179) of individuals declared sexual intercourse in the previous month and were considered. Among those who completed the questionnaire, 36% scored below the FSFI total cut-off, indicating increased risk of SD. The most frequently impaired domains were desire (56.4%) and lubrication (52.1%). The correlation between age and total FSFI score was significant (*P* = .008), as well as menopausal women obtained lower FSFI scores (*P* = .0004).

**Clinical Implications:**

Age and menopausal status are substantial factors influencing sexual functionality.

**Strengths and Limitations:**

This study is limited by its reliance on self-reported data and a sample size that may be insufficient for detecting subtle effects. However, it leverages the well-validated FSFI tool and benefits from trusted questionnaire administration by HIV healthcare providers.

**Conclusion:**

Sexual dysfunction in menopausal WLWH has a high prevalence (36%). Interestingly, around 67% of women declined to investigate and deal with sexual issues.

## Background

Thanks to the widespread use of antiretroviral therapy (ART), HIV has evolved into a manageable chronic condition, with effective treatment leading to viral suppression and sexual non-transmissibility.

In Italy, approximately 140 000 people are living with HIV. In 2023, women accounted for 24% of new HIV diagnoses, with a high percentage receiving ART and achieving viral suppression^1^

Sexual health is a crucial component of overall sexual health and plays a significant role in determining quality of life.^2^^,^^3^According to the World Health Organization (WHO), sexual health includes a state of holistic well-being, encompassing physical, emotional, mental, and social dimensions concerning sexuality.^4^^,^^5^It requires a positive and respectful approach to sexuality and sexual relationships, as well as the possibility of having pleasurable and safe sexual experiences, free from coercion, discrimination, and violence.^3^

Women living with HIV (WLWH) represent a unique population in this context, as they may face specific challenges related to sexual health. Despite the success of ART in improving life expectancy and quality of life, WLWH may still encounter biological, psychological, and social barriers to sexual well-being.^4^^,^^6^These include stigma, concerns about transmission, treatment side effects, hormonal fluctuations, and relationship dynamics.

The delineation of sexual dysfunction (SD), particularly in the context of women, poses a challenge in terms of objective description and measurement, given the limited availability of gender-specific validated assessment tools.^7^

Female sexual dysfunction (FSD) is defined by the WHO as a person’s inability to participate in a sexual relationship as they would wish.^8^According to the Diagnostic and Statistical Manual of Mental Disorders (DSM-5), a diagnosis of SD disorder requires symptoms to persist for at least six months and to cause significant distress to the individual.^9^^,^^10^

FSD encompasses several domains of sexual function, including reduced sexual desire, arousal difficulties, problems with lubrication, orgasmic dysfunction, pain during intercourse, and low sexual satisfaction.^7^^,^^10^These dimensions are commonly assessed using validated tools such as the Female Sexual Function Index (FSFI), a 19-item questionnaire that evaluates six domains: desire, arousal, lubrication, orgasm, satisfaction, and pain.^7^

Sexual dysfunction is more prevalent among people living with HIV than in the general population.^6^^,^^11^^,^^12^Among WLWH, several studies have highlighted a high prevalence of sexual difficulties. For example, rates of FSD as high as 89.2% have been reported in Nigeria,^13^52% in India,^14^47% of sexual dissatisfaction in England,^11^and 36% of low sexual desire in a Brazilian cohort.^15^However, literature on sexual satisfaction, arousal, and dysfunction in WLWH remains limited, with most studies focusing on risky sexual behaviors rather than on sexual health as a quality-of-life concern.^13^

There is also a documented gap in the clinical attention given to sexual function in WLWH. Bell et al. reported that 61% of HIV specialists rarely or never assess sexual function, and only 2.5% actively address it during consultations.^11^As a result, the true burden of SD in this population may be underestimated, and opportunities for early intervention missed.

FSD in WLWH is influenced by biological, psychological, socio-cultural, and relational factors. Menopausal status, aging, and comorbid conditions such as cardiovascular disease, diabetes, and mental health disorders can all contribute to reduce sexual functioning.^13^Moreover, symptoms of chronic illness and side effects of antiretroviral drugs may further impact sexual health.^13^^,^^15^^,^^16^

Therefore, this study aimed to investigate the prevalence of female SD symptoms and the associated risk factors in a cohort of WLWH receiving care at a tertiary university hospital in Northern Italy.

## Methods

### Study design

This monocentric cross-sectional study was conducted at the ASST (Azienda Socio Sanitaria Territoriale) Spedali Civili of Brescia, northern Italy, between April 2023 and August 2023. Data were collected using a pen-and-paper anonymous questionnaire. During the completion of the questionnaire, healthcare professionals were available to provide support throughout the process.

### Participants

Participants were recruited during routine visits at the HIV outpatient clinic of our hospital. The study included all cisgender WLWH who were 18 years of age or older who attended a routine visit at the HIV outpatient clinic of our hospital.

Exclusion criteria included a lack of proficiency in Italian or English, which prevented completion of the FSFI questionnaire.

For the primary analysis, only participants who reported engaging in sexual intercourse within the past month were included. Women who did not report sexual activity during this period were included exclusively in the analysis of the “desire” domain.

### Data collection

Sexual dysfunction was evaluated using the FSFI questionnaire, administered in alignment with current European AIDS Clinical Society guidelines.

The questionnaire was available in both the original English version and a validated Italian translation.

### Measures

Data were collected using the FSFI, a validated 19-item questionnaire that assesses 6 key domains of sexual function:

- Desire
- Arousal
- Lubrication
- Orgasm
- Satisfaction
- Pain

Each item is scored on a scale from 0 to 5, with higher scores indicating better sexual function.

The total FSFI score ranges from 2 to 36, and a cutoff score of ≤26.55 has been established to identify individuals at risk of SD. Completion of the questionnaire required approximately 10 minutes.

### Clinical and virological data

Clinical and virological data were collected during the same clinical visit in two phases. First, the attending physician completed the clinical section of the questionnaire together with the participant, using information retrieved from the patient’s electronic medical record. This included comorbidities (eg, cardiovascular diseases, diabetes, and psychiatric disorders), use of antidepressant or antipsychotic medications, menopausal status, and educational attainment. Self-reported information was used to complement or verify data from the chart, such as symptoms, partner status, or recent medication changes. In case of any discrepancies between patient report and the medical record, the version documented in the electronic medical record was considered the reference.

In the second phase, the FSFI questionnaire was self-administered by the participant in a private setting, without physician involvement.

Regarding HIV-related parameters, viro-immunological data were extracted from the hospital’s electronic medical records, including:

- Plasma HIV-RNA levels (to determine whether the patient had undetectable viremia, defined as <20 copies/mL)
- CD4+ T-cell count

All collected data were de-identified and securely stored in a REDCap© database.

### Ethics

The study protocol was approved by the ASST Spedali Civili Ethics Committee (protocol NP 5184) and conducted in accordance with the ethical standards of the Helsinki Declaration (1975, revised in 2013). Written informed consent was obtained from each subject.

### Statistical analysis

Categorical variables were expressed as number (percentage), and continuous variables were expressed as mean and SD. Categorical data were compared by using the Student’s *t*-test, χ^2^test or the Fisher’s exact test, as appropriate. Statistical analysis was conducted with GraphPad Prism software.

The study population was stratified into two groups based on the total FSFI score: participants with a score of ≥26.55 and those scoring below this threshold. As no established cutoffs exist for the individual domains, the median score for each domain was calculated, and participants were divided into two groups: those scoring above or equal to the median and those scoring below it. The influence of demographic characteristics, menopausal status, and comorbidities was then examined for each domain individually.

## Results

**Figure 1:**
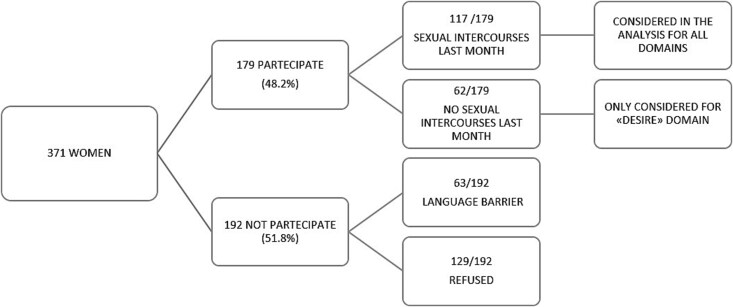
Illustrations of the rectal cavity of cisgender women and cisgender men divided into 4 unique selectable regions: Superficial anterior rectum, superficial posterior rectum, deep anterior rectum, deep posterior rectum.

A total of 371 women were initially invited to participate in the study. Of these, 179 women (48.2%) met the inclusion criteria and consented to participate. As illustrated in Figure 1, the primary reasons for exclusion from the study included language barrier (32.8%) and rejection to participate (67.2%). Among the participants, 117 women (65.4%) reported engaging in sexual intercourse during the preceding month. The remaining 62 women (34.6%) were included exclusively in the analysis of the “desire” domain of the FSFI questionnaire.

The study population predominantly comprised individuals of Italian nationality (88,3%), with a mean age of 53.1 years (± 9.8). A notable proportion of the cohort (64.8%) consisted of women experiencing physiological or post-surgical menopause, with an average duration of menopause of 9.2 years (± 6.9). Regarding relationship status, 131 women reported being in a stable relationship, 43 women reported having an occasional partner, while only 5 women indicated that they were stably without a partner (Table 1).

**Table 1:** Demographic characteristics of the population of women with HIV who responded to the questionnaire.

**Age**	
Mean age (years), mean (SD)	53.1 (±9.8)
Median age (years), median (range)	54 (22-77)
**Nationality**	
Italian, *n*(%)	158 (88.27%)
Not Italian, *n*(%)	21 (11.73%)
• European region, *n*(%)	9 (42.86%)
• African region, *n*(%)	10 (47.62%)
• Region of the Americas, *n*(%)	2 (9.52%)
**Menopause**	
Yes, *n*(%)	116 (64.8%)
No, *n*(%)	63 (35.2%)
Menopausal length (years), mean(SD)	9.2 (±6.9)
**Partner**	
Regular, *n*(%)	131 (73.18%)
Occasional, *n*(%)	43 (24.02%)
No partner, *n*(%)	5 (2.79%)

The analysis of the FSFI questionnaire revealed that 35.9% of participants scored below 26.55, suggesting a higher risk of female SD. The mean score obtained was 28.3 (± 4.8). Women living with HIV who were at higher risk of SD were significantly older than those with lower risk of SD (*P* = .008). Additionally, the analysis indicates an association between menopausal status and the prevalence of SD. Specifically, 78.6% of women reporting SD were menopausal, compared to 42.7% of those without reported sexual issues. However, the duration of menopause did not show a significant correlation, as the number of menopausal years was nearly identical in both groups. Our findings show that comorbidities, partner status, or nationality did not significantly influence overall FSFI score (Table 2).

**Table 2:** Comparison between the population with and without sexual dysfunction, as calculated by the FSFI questionnaire using Student’s *t*-test, χ^2^test or the Fisher exact test.

**No FSD**		**FSD**	***P*-value**
Women, *n*(%)	75 (64.1%)	42 (35.9%)	
Age, mean	48.9 (±6.7)	54 (±9.9)	.008
**Nationality**			
Italian	65 (86.7%)	36 (85.7%)	.6544
Other	10 (13.3%)	6 (14.3%)	
**Menopause**			
Yes, *n*(%)	32 (42.7%)	33 (78.6%)	.0004
No, *n*(%)	43 (57.3%)	9 (21.4%)	
Menopausal length (years), mean (SD)	8.1 (±5.1)	8.9 (±5.6)	.5958
**Partner**			
Permanent, *n*(%)	68 (90.7%)	40 (95.2%)	1
Occasional/No partner, *n*(%)	7 (9.3%)	2 (4.8%)	
**Comorbidity**			
Yes, *n*(%)	63 (84%)	33 (78.6%)	.6292
No, *n*(%)	12 (16%)	9 (21.4%)	
**Psychiatric therapy**			
Yes, *n*(%)	10 (13.3%)	8 (19.4%)	.5791
No, *n*(%)	65 (86.7%)	34 (80.6%)	
**HIV-RNA undetectable**			
Yes, *n*(%)	69 (92%)	41 (97.6%)	.219
No, *n*(%)	6 (8%)	1 (2.4%)	
CD4+ (cells/mcL), mean	860.5(±325.1)	851.1(±401.3)	.89

Abbreviations: FSD, female sexual dysfunction. FSFI, Female Sexual Function Index.

Participants were stratified into two groups (above and below the median) for each FSFI domain to assess associations with sociodemographic and clinical variables. The domains most frequently affected were desire (56.4%) and lubrication (52.1%).

**Figure 2:**
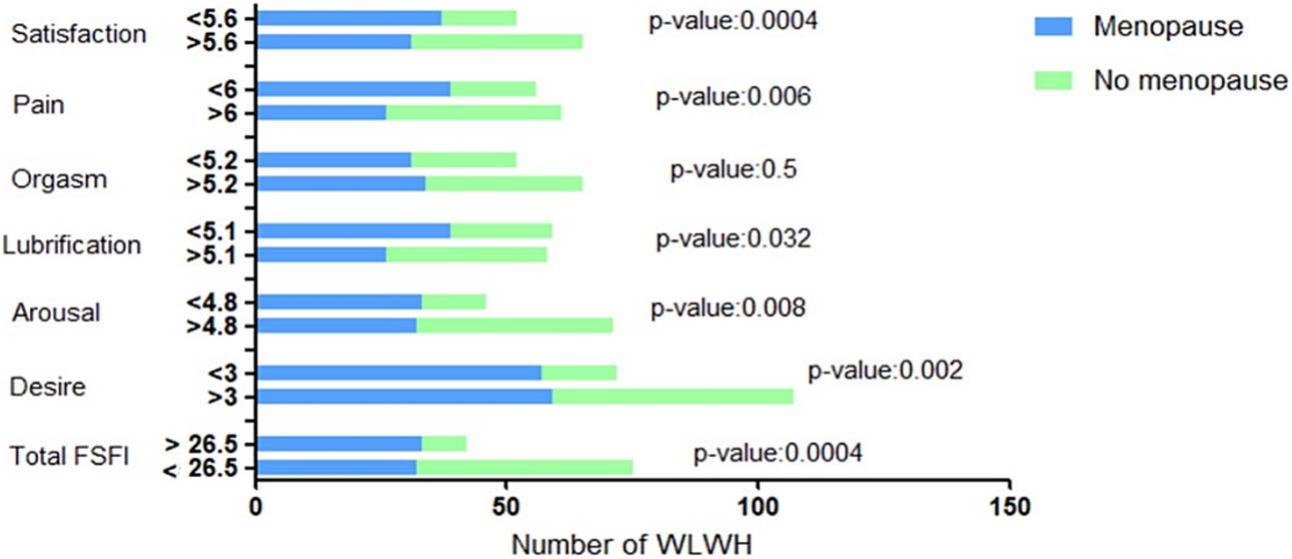
Mean percentages of cisgender women and cisgender men respondents who designated each region pleasurable. Darker shades of green correlate with higher likelihood of selection.

Lower scores in the desire domain were significantly associated with older age (*P* = .002), menopausal status (*P* = .0017), longer duration of menopause (*P* = .0017), and the use of antidepressant or antipsychotic medications (*P* = .0127) (Table 3, Figure 2).

**Table 3:** Factors influencing each domain. (Median scores: desire = 3; arousal = 4.8; lubrification = 5.1; orgasm = 5.2; pain = 6; satisfaction = 5.6).

**Desire**				**Arousal**			**Lubrification**			**Orgasm**			**Pain**			**Satisfaction**		
**score ≥ 3**		**score < 3**	***P*- value**	**Score ≥ 4.8**	**Score < 4.8**	***P*-value**	**Score ≥ 5.1**	**Score < 5.1**	***P*-value**	**score ≥ 5.2**	**Score < 5.2**	***P*-value**	**Score ≥ 6**	**Score < 6**	***P*-value**	**score ≥ 5.6**	**Score > 5.6**	***P*-value**
**Women number (%)**	107 (59.8%)	72 (40.2%)		71 (60.7%)	46 (39.3 %)		58 (49.6%)	59 (50.4%)		65 (55.6%)	52(44.6%)		61 (52.1%)	56 (47.9%)		68 (58.1%)	49 (41.9%)	
**Age (mean)**	51.3 (±10-3)	55.8 (±8.5)	.002	50 (±9.3)	51.9 (±10.6)	.3104	49.5 (±10)	52 (±10)	.1815	51.4 (±9)	49.8 (±11.3)	.3923	49.4 (±9.9)	52.1 (±10.1)	.1415	50.1 (8.7)	51.6 (±11.5)	.4479
**Nationality**																		
Italian	95	63	.99	62	39	.9082	50	51	.8163	55	48	.2582	54	47	.6502	64	44	.4884
Other	12	9		9	7		8	8		10	6		7	9		4	5	
**Menopause**																		
Yes	59	57	.0017	32	33	.0082	26	39	.0332	34	31	.5464	26	39	.0059	31	34	.0179
No	48	15		39	13		32	20		31	21		35	17		37	15	
**Menopausal length (years)**	4.6	7.9	.0017	4	5.9	.1039	3.7	5.7	.0752	4.5	5.1	.6297	4.1	5.5	.2159	3.7	6.3	.0202
**Partner**																		
Permanent	90	41	.3138	65	43	>.999	55	55	>.99	58	50	.2952	54	54	.1662	63	45	.8498
Occasional/no	4	0		6	3		3	4		7	2		7	2		5	4	
**Comorbidity**																		
Yes	88	53	.23	59	37	.9044	45	51	.314	54	42	.9356	51	45	.8287	55	41	.8498
No	19	19		12	9		13	8		11	10		10	11		13	8	
**Psychiatric therapy**																		
Yes	15	22	.0127	11	7	.8244	46	52	.2968	10	8	.7965	11	8	.7656	9	10	.4331
No	92	50		60	39		12	7		55	44		50	48		59	39	
**HIV-RNA undetectable**																		
Yes	101	63	.17	66	44	.702	54	56	.7168	62	48	.6983	56	54	.4418	63	47	.6975
No	6	9		5	2		4	3		3	4		5	2		5	2	
**CD4+ (Mean)**	849.7	811.7	.1554	879.2	823.1	.1554	847.65	866.6	.7701	857.1	857.1	1	874.4	838.3	.5829	871.8	836.7	.5974

Menopausal status was also significantly associated with lower scores in the arousal (*P* = .0053) and lubrication (*P* = .0045) domains (Table 3, Figure 2). In the pain (*P* = .0004) and satisfaction (*P* = .0318) domains, both menopausal status and longer duration of menopause were again associated with worse outcomes (Table 3, Figure 2). No significant associations were identified in the orgasm domain.

## Discussion

This was a cross-sectional study that aimed to investigate the prevalence of SD and its associated factors in a cohort of WLWH receiving care at a large tertiary university hospital in Northern Italy, with menopausal status, age, and use of psychotropic medications emerging as the main contributors.

The analysis of the FSFI questionnaire revealed that 35.9% of participants scored below 26.55, suggesting a higher risk of FSD, in accordance with widely used FSFI cut-off scores.

The study describes the relationship between menopausal status and SD in a group of WLWH as described in general population^17^with a notably higher percentage of menopausal WLWH reporting issues compared to their non-menopausal counterparts.

Our study revealed that, out of 179 participants, 117 reported having engaged in sexual intercourse in the preceding month, of whom 35.9% were classified as at increased risk of SD based on their FSFI score. When compared with findings from other studies, our results suggest that the sexual quality of life in our cohort is relatively better than in other populations.^13^^,^^18^^,^^19^However, it remains notable that over one-third of the women analyzed still experience SD. For instance, a study conducted by Agaba et al. in 2017, which assessed sexual functioning in a cohort of WLWH receiving care at a hospital in Nigeria between 2013 and 2014, utilized the FSFI questionnaire and reported different results. In that investigation, 89.2% of the women exhibited SD.^13^Another investigation conducted among WLWH in New York during the period of 2006 to 2007 reveals a notable contrast in FSFI scores. The mean FSFI score reported stands at 13.8 (± 12.7), markedly lower when juxtaposed with our cohort’s findings (mean 28.3± 4.8).^19^This disparity is further echoed in Florence’s 2004 study, wherein the FSFI questionnaire yields a median score of 25.2.^18^Conversely, in our study, participants exhibit a median score of 29.1, highlighting a deviation in sexual functioning compared to previous research outcomes.^18^It is conceivable that the observed amelioration in outcomes within our cohort could be partially attributed to the temporal disparity between studies, spanning approximately 10-20 years, suggesting that the attenuation of social taboos surrounding sexuality over time may have played a contributory role in the observed enhancements. To substantiate this conjecture, it is noteworthy to mention a study conducted in Denmark during the period spanning 2021-2022. In this investigation, it was observed that the prevalence of SD among WLWH, as diagnosed by FSFI, amounted to 33.3%.^20^This figure closely mirrors the results obtained in the present study, reinforcing the consistency of our findings.

Our findings reveal a high prevalence SD among WLWH as described in woman with other chronic diseases^21-24^and a significant correlation between SD and age similar to what happens in the general population.^25^However, what stands out prominently is how menopause emerges as the most relevant factor, both in total scores and in individual domains, independently of the chronic underlying pathology.^26^^,^^27^

The 2010 study conducted by Wilson et al. identified correlations with psychiatric comorbidities and SD.^19^We did not observe this relation in our sample; however, we found a significant association between reduced sexual desire and the use of antidepressant therapy. This aligns with evidences from the most recent literature which suggest the role of anti-psychiatric drugs as a possible contributing factor to SD.^28^^,^^29^

Despite the limitation of our study of not conducting a direct comparison between WLWH and HIV negative women, existing literature highlights menopause as a highly influential factor on sexual functionality even in general population.^20^^,^^30^^,^^31^This is demonstrated by a Spanish study published in 2020, where menopausal symptoms are associated with lower scores both overall and in the domains of lubrication, satisfaction, arousal, and orgasm.^32^In our study, menopause also influenced the desire domain, while it does not appear to be associated with difficulties in orgasm.

The role of comorbidities, including diabetes, dyslipidemia, atherosclerosis, and renal insufficiency, in the pathogenesis of SD is extensively described in general female population.^16^A study conducted in India on HIV negative women in 2022 found that women with diabetes mellitus scored lower on the FSFI questionnaire compared to the control group.^33^An analysis conducted through the third National Survey of Sexual Attitudes and Lifestyles (Natsal-3) among British HIV- negative individuals aged 16-74, interviewed between 2010 and 2012, reveals an association between SD and cardiovascular conditions such as hypertension, myocardial infarction, stroke, diabetes, pulmonary, neurological, and gynecological disorders.^34^The correlation with hypertension is further supported by a population-based study demonstrating that FSFI scores differed significantly between normotensive and hypertensive individuals. The difference in FSFI scores was also statistically significant when comparing hypertensive patients undergoing medical treatment with those not receiving therapy.^35^

In our investigation, the presence or absence of concurrent pathologies like cardiovascular diseases, diabetes, oncologic diseases and psychological disorders exhibited no discernible impact on the questionnaire outcomes, a trend consistent with the findings elucidated in Wilson et al.^19^Conversely, within Agaba et al.’s study framework, afflictions such as diabetes mellitus and systemic sclerosis were mentioned as correlating with diminished aggregate scores.^13^Lastly, the absence of association between HIV-related factors as CD4+ t-cell count or HIV-RNA values, indicates that HIV infection alone seems not to promote SD in our population. The majority of the women in our cohort had well-controlled HIV infection, with strict adherence to ART and undetectable viral loads, which may have contributed to the lack of association observed. The association between uncontrolled HIV infection and FSD is well established in the literature, as more severe HIV-related symptoms and low ART adherence have been linked to decreased sexual function, including lower levels of libido, pleasure, and satisfaction. This suggests that adequate control of the infection through ART may mitigate some of the negative effects on sexual function.^16^

Our results should be seen in light of some limitations. One of the primary limitations of this study is the reliance on self-reported data, which may introduce bias due to inaccuracies in ‘recollection of participants or willingness to disclose sensitive information, particularly regarding sexual function. The use of a single, standardized questionnaire (FSFI) may not fully capture the complexity of SD, especially in a population with diverse cultural and socio-economic backgrounds. Another important limitation is the absence of a formal assessment of sexual distress. Although the FSFI is a validated tool for evaluating sexual function symptoms, it does not capture the psychological distress required to meet diagnostic criteria for female SD disorders, as defined by the DSM-5. Therefore, our findings reflect symptoms and risk indicators, but cannot be interpreted as clinical diagnoses. This distinction has been emphasized in recent literature highlighting the central role of distress in diagnosing female SD, particularly during menopause.^36^Additionally, the sample size, while sufficient for preliminary analysis, may not be large enough to detect more subtle effects or interactions between variables. Finally, the results of a cross-sectional study are often limited by a selection bias. A key limitation of this study lies in the relatively low response rate: only 48.2% of eligible women agreed to complete the FSFI questionnaire. This reluctance may reflect a broader discomfort in discussing sexual health, even in the clinical setting. In comparison, a European study conducted between 2000 and 2002 reported a higher response rate (77%) among WLWH, possibly due to greater linguistic accessibility: questionnaires were offered in six languages, while in our center, 63 women were excluded due to language barriers.^18^This suggests that inclusivity in research materials may impact engagement. Reluctance to seek medical assistance for sexual problems, particularly among aging women, has also been emphasized in recent international guidelines, which call for increased awareness among healthcare providers.^37^Additionally, participants were informed about the study during their routine clinical visits and asked to decide at that time whether to participate. This approach, although common in clinical settings, may have influenced the response rate, as some women may have felt unprepared or uncomfortable making an immediate decision. Another limitation is the statistical analysis, which primarily focuses on bivariate analysis. Inferential statistical methods were not applied, which may have constrained the ability to draw more robust conclusions about the relationships between variables. Future studies should incorporate inferential analysis to provide a deeper understanding of these associations. Despite these limitations, the study has several strengths. The use of the FSFI, a well-validated and widely recognized tool for assessing sexual function, provides a robust and standardized method for data collection; moreover, questionnaires were administered by the patients’ regular HIV care providers, who have been following them in a long-term clinical setting.

The study also benefits from a diverse sample, including participants from different nationalities and menopausal statuses, which adds relevance to the findings and may support their applicability to similar clinical settings. Moreover, the thorough statistical analysis, including the comparison of both total scores and individual domain scores, allows for a nuanced understanding of the factors influencing sexual function in the study population. Lastly, the study’s focus on the impact of menopausal status and comorbidities on sexual function adds valuable insights to the existing literature, particularly in the context of WLWH.

In terms of clinical implications, our study underscores the urgent need for further investigation into SD among WLWH. Despite the relatively high prevalence observed in our cohort, sexual health issues appear to be under-addressed in clinical practice. This may partly be due to the social stigma and lack of open dialog surrounding sexual health, resulting in limited screening and management of these conditions.

The findings suggest that healthcare providers should incorporate routine screening for SD during consultations with WLWH. Early identification and intervention could lead to more tailored and effective management strategies, ultimately improving overall quality of life. Furthermore, given those factors such as menopausal status significantly impact sexual functioning, there is a pressing need for multidisciplinary approaches that address both the biological and psychosocial aspects of sexual health in this population.

## Conclusions

In conclusion, it can be asserted that SD among WLWH constitutes a significant issue that receives inadequate attention from the affected women themselves, as evidenced by the considerable proportion of subjects who declined to respond to the questionnaire. Age emerged as a substantial factor influencing sexual functionality, with menopausal status playing a predominant role, aligning with existing literature and the demographics of healthy women. It is also evident that lubrication is the component of sexual function most affected.

## Supplementary Material

FSFI_ENG_qfaf038
