# Supplementary material for: Female sexual dysfunction: prevalence and risk factors in a cohort of women living with HIV in Italy
Source: Sex Med. 2025 May 19;13(2):qfaf038. doi: 10.1093/sexmed/qfaf038 (PMC12090195; doi:10.1093/sexmed/qfaf038)
Supplement: FSFI_ENG_qfaf038 [file fsfi_eng_qfaf038.pdf]

Female Sexual Function Questionnaire

The World Health Organization defines sexual health as "a state of physical, emotional, mental, and social well-being in relation to sexuality; it is not merely the absence of disease, dysfunction, or infirmity. It requires a positive, respectful approach to sexuality and the possibility of having safe and pleasurable sexual experiences free from coercion, discrimination, and violence."

In this regard, sexual well-being is recognized not only as a component of general health but as a condition of emotional, physical, mental, and social satisfaction and serenity. Every woman may feel the need to seek advice and, if necessary, undergo a diagnostic and therapeutic path to understand the causes and identify issues related to possible sexual dysfunction.

The following questionnaire investigates the Female Sexual Function Index (FSFI) and is developed to assess issues such as sexual arousal, orgasm, satisfaction, and pain.

The following questions concern the sexual feelings and behaviors over the past 4 weeks. When answering the questions, please consider the following definitions:

- Sexual activity may include touching (petting), masturbation, and vaginal penetration.
- Sexual intercourse refers to penile-vaginal penetration.
- Sexual stimulation includes activities such as masturbation, sexual play with partners, or erotic fantasies.
- Sexual desire or interest refers to the feeling of seeking a sexual experience, willingness to accept sexual stimulation from a partner, or thinking or fantasizing about having sex.

Please choose only one answer for each question.

Do you agree to participate in this questionnaire?
YEAR OF BIRTH
COUNTRY OF BIRTH

Level of education achieved:

- None
- Elementary School Diploma
- Middle School Diploma
- High School Diploma
- University Degree
- Don't know/No answer

Are you menopausal?

- YES
- NO

If YES, for how many years?
Do you have a partner?

- NO

- Only a fixed partner
- Only an occasional partner

Do you have any of the following chronic conditions?

- NO
- Diabetes
- Cancer-related conditions
- Cardiovascular diseases
- Psychological/behavioral disorders
- Don't know/No answer

Have you taken antidepressants or psychotropic drugs in the last year?

- YES
- NO
- Don't know

---

1. **In the last 4 weeks, how often have you experienced sexual desire or interest?**

- Almost always or always
- Often (more than half the time)
- Sometimes (about half the time)
- Rarely (less than half the time)
- Almost never or never
- Don't know/No answer

2. **In the last 4 weeks, how would you rate your level of sexual desire/interest?**

- Very high
- High
- Medium
- Low
- Very low or absent
- Don't know/No answer

3. **In the last 4 weeks, how often have you experienced pleasure during sexual activity/intercourse?**

- Almost always or always
- Often (more than half the time)

- Sometimes (about half the time)
- Rarely (less than half the time)
- Almost never or never
- Don't know/No answer

4. **In the last 4 weeks, how would you rate your level of arousal during sexual activity/intercourse?**

- Very high
- High
- Medium
- Low
- Very low or absent
- Don't know/No answer

5. **In the last 4 weeks, how comfortable (secure) did you feel during sexual activity/intercourse?**

- No sexual activity
- Very secure
- Secure
- Moderately secure
- Not very secure
- Not at all secure
- Don't know/No answer

6. **In the last 4 weeks, how often were you satisfied with your pleasure (arousal) during sexual activity/intercourse?**

- No sexual activity
- Almost always or always
- Often (more than half the time)
- Sometimes (about half the time)
- Rarely (less than half the time)
- Almost never or never
- Don't know/No answer

7. **In the last 4 weeks, how often did you become lubricated (wet on your own due to arousal) during sexual activity/intercourse?**

- No sexual activity

- Almost always or always
- Often (more than half the time)
- Sometimes (about half the time)
- Rarely (less than half the time)
- Almost never or never
- Don't know/No answer

8. **In the last 4 weeks, how difficult was it to become lubricated (wet) during sexual activity/intercourse?**

- No sexual activity
- Extremely difficult or impossible
- Very difficult
- Difficult
- Slightly difficult
- Not difficult
- Don't know/No answer

9. **In the last 4 weeks, how often did you maintain lubrication (stay wet) until the end of sexual activity/intercourse?**

- No sexual activity
- Almost always or always
- Often (more than half the time)
- Sometimes (about half the time)
- Rarely (less than half the time)
- Almost never or never
- Don't know/No answer

10. **In the last 4 weeks, how difficult was it to maintain lubrication (stay wet) until the end of sexual activity/intercourse?**

- No sexual activity
- Extremely difficult or impossible
- Very difficult
- Difficult
- Slightly difficult
- Not difficult
- Don't know/No answer

11. **In the last 4 weeks, how often did you reach orgasm during sexual stimulation/intercourse?**

- No sexual activity
- Almost always or always
- Often (more than half the time)
- Sometimes (about half the time)
- Rarely (less than half the time)
- Almost never or never
- Don't know/No answer

12. **In the last 4 weeks, how difficult was it to reach orgasm during sexual stimulation/intercourse?**

- No sexual activity
- Extremely difficult or impossible
- Very difficult
- Difficult
- Slightly difficult
- Not difficult
- Don't know/No answer

13. **In the last 4 weeks, how satisfied were you with your ability to reach orgasm during sexual activity/intercourse?**

- No sexual activity
- Very satisfied
- Moderately satisfied
- Somewhat satisfied
- Not very satisfied
- Not satisfied
- Don't know/No answer

14. **In the last 4 weeks, how often did you experience pain DURING vaginal penetration?**

- No attempt at intercourse
- Almost always or always
- Often (more than half the time)
- Sometimes (about half the time)
- Rarely (less than half the time)

- Almost never or never
- Don't know/No answer

15. **In the last 4 weeks, how often did you experience pain AFTER vaginal penetration?**

- No attempt at intercourse
- Almost always or always
- Often (more than half the time)
- Sometimes (about half the time)
- Rarely (less than half the time)
- Almost never or never
- Don't know/No answer

16. **In the last 4 weeks, how would you rate your level of pain or discomfort during or after vaginal penetration?**

- No attempt at intercourse
- Very high
- High
- Medium
- Low
- Very low or absent
- Don't know/No answer

17. **In the last 4 weeks, how satisfied are you with the emotional intimacy with your partner during sexual activity?**

- No sexual activity
- Very satisfied
- Moderately satisfied
- Somewhat satisfied
- Not very satisfied
- Not satisfied
- Don't know/No answer

18. **In the last 4 weeks, how satisfied are you with the sexual rapport with your partner?**

- No sexual activity
- Very satisfied
- Moderately satisfied

- Somewhat satisfied
- Not very satisfied
- Not satisfied
- Don't know/No answer

19. **In the last 4 weeks, how satisfied are you with your overall sexual life?**

- No sexual activity
- Very satisfied
- Moderately satisfied
- Somewhat satisfied
- Not very satisfied
- Not satisfied
- Don't know/No answer
